# Trypanothione reductase overexpression increases the infectivity of *Leishmania tarentolae* through improved redox homeostasis and increased resistance to oxidative stress

**DOI:** 10.3389/fcimb.2026.1824711

**Published:** 2026-08-17

**Authors:** Myslene Soares Fonseca, Rodrigo Prado Rodrigues Miranda, Job Domingos Filho Inácio, Tania Zaverucha Do Valle, Elmo Eduardo Almeida-Amaral

**Affiliations:** 1 Laboratório de Bioquímica de Tripanosomatídeos, Instituto Oswaldo Cruz, Fundação Oswaldo Cruz, Rio de Janeiro, Brazil; 2 Laboratório de Epidemiologia e Malformações Congênitas, Instituto Oswaldo Cruz, Fundação Oswaldo Cruz, Rio de Janeiro, Brazil; 3 Laboratório de Protozoologia, Instituto Oswaldo Cruz, Fundação Oswaldo Cruz, Rio de Janeiro, Brazil

**Keywords:** *Leishmania*, trypanothione reductase, infectivity, oxidative stress, antioxidant defense, host‒parasite interaction

## Abstract

Leishmaniasis is a neglected tropical disease that imposes a significant global health burden. To establish persistent infections, parasites must overcome oxidative stress generated by host immune responses, a process for which the parasite-specific enzyme trypanothione reductase (TR) is critical. While TR has been implicated in infectivity, its precise role in governing infectivity remains incompletely understood. This study aimed to investigate the hypothesis that TR activity is a key determinant of parasite infectivity. We first compared TR activity between pathogenic *Leishmania infantum* and nonpathogenic *L. tarentolae* and found that *L. infantum* exhibits 5.7-fold higher TR activity than *L. tarentolae*. Structural analysis revealed significant amino acid substitutions (Asp116Glu and Lys240Gln) in *L. tarentolae* TR that likely account for the observed reduction in TR activity. To functionally validate the role of TR, we generated *L. tarentolae* lines overexpressing TR. Compared with wild-type *L. tarentolae*, these mutants exhibit 6-fold higher enzyme activity (p < 0.0001) and significantly increased resistance to hydrogen peroxide (IC_50_: 515 µM vs. 425 µM in the wild type; p < 0.0001). Consequently, TR-overexpressing parasites show a dramatic increase in infectivity in murine peritoneal macrophages (8-fold at 24 hours; 78-fold at 72 hours). Most notably, *in vivo* infection experiments in BALB/c mice (n=5 per group) revealed that compared with wild-type parasites, TR-overexpressing parasites result in a 3.3-fold higher liver parasite load. These findings establish that TR activity is an important infectivity-associated factor that contributes substantially to parasite infectivity by increasing antioxidant capacity. Our results strongly support TR as a high-priority drug target, suggesting that its inhibition represents a promising strategy for developing novel drugs against leishmaniasis and other kinetoplastid infections.

## 1 Introduction

Leishmaniasis, caused by protozoan parasites of the genus *Leishmania*, is a neglected tropical disease (NTD) that imposes a substantial global health burden. Infectious agents of the genus *Leishmania* have a digenetic life cycle comprising two main forms: promastigotes, which are found in the midgut of the sandfly, and amastigotes, which reside in mononuclear phagocytic cells of the vertebrate host after infection (Silva-Moreira et al., 2025). The transition of the parasite between these distinct environments and its continuous exposure to both invertebrate and vertebrate immune responses require sophisticated biochemical and molecular adaptations. Among these adaptations, antioxidant control is essential for parasite survival and for the establishment of infection.

Upon infection of a mammalian host, intracellular parasites such as *Leishmania* species face a formidable challenge: the generation of reactive oxygen species (ROS) by macrophages as a primary antimicrobial defense mechanism. This oxidative burst is among the most potent resources in the innate immune arsenal against intracellular pathogens. Consequently, efficient antioxidant systems are central to parasite persistence within host cells and to the development of chronic infection. In kinetoplastid parasites, this antioxidant defense is orchestrated by the trypanothione system. Within this system, trypanothione reductase (TR) sustains the reducing capacity required for parasite adaptation to the hostile environment of infected macrophages (Roy et al., 2025).

This parasite-specific defense mechanism stands in stark contrast to the system found in their hosts. In mammals, redox homeostasis is regulated by glutathione and glutathione reductase. However, kinetoplastid parasites such as *Leishmania* do not express or synthesize glutathione reductase. Instead, *Leishmania* protozoa synthesize a parasite-specific antioxidant molecule, trypanothione, and express the enzyme trypanothione reductase (TR) (EC 1.8.1.12). TR is a NADPH-dependent enzyme that catalyzes the reduction of oxidized trypanothione, thereby regenerating the reduced form required for antioxidant defense (Fairlamb and Cerami, 1992; Krauth-Siegel et al., 2003).

Experimental evidence from several kinetoplastid models supports a functional link between TR activity and parasite infectivity. A mutant *Trypanosoma brucei* strain with low TR activity was found to be unable to establish infection in mice and to exhibit markedly increased susceptibility to hydrogen peroxide produced by the host immune system (Krieger et al., 2000). Similarly, genetic knockout of TR in *Leishmania donovani* resulted in significantly reduced parasite survival within macrophages and decreased infection rates *in vivo*. Conversely, overexpression of TR in *Trypanosoma cruzi* increased parasite infectivity, demonstrating not only that TR plays a housekeeping function but also that TR activity is a critical determinant of parasite infectivity and survival under oxidative stress (Dumas et al., 1997; González-Chávez et al., 2019). Together, these findings support that TR contributes to infection-associated phenotypes across kinetoplastid parasites and that changes in TR activity may influence their capacity to persist in mammalian hosts.

Although TR has been implicated in parasite survival and infection, its specific contribution can be further dissected using the unique model organism, *Leishmania tarentolae*. This parasite naturally infects reptiles (lizards) and is nonpathogenic to mammalian hosts (Klatt et al., 2019). Notably, *L. tarentolae* exhibits low or no expression of genes encoding components of the antioxidant system, including those encoding proteins involved in the trypanothione system (Raymond et al., 2012). This ‘natural knockout’ phenotype provides an exceptional opportunity to investigate the specific role of TR in infectivity. However, the current data do not provide sufficient information on the role of TR in the infectivity of *L. tarentolae*, nor do they address whether restoring TR activity in this nonpathogenic species could confer pathogenic traits.

A direct comparison between the pathogenic species *Leishmania infantum* and the nonpathogenic species *L. tarentolae* therefore provides an opportunity to examine how TR-dependent antioxidant capacity contributes to host adaptation. *L. infantum* is a mammalian pathogen that causes visceral leishmaniasis, characterized by high parasite loads in the liver and spleen, whereas *L. tarentolae* remains nonpathogenic even when it is experimentally introduced into mammalian hosts. This natural contrast provides an ideal experimental framework for delineating the molecular basis of infection-associated phenotypes. If TR activity is indeed a critical infectivity-associated factor, then restoring TR function in *L. tarentolae* should increase its ability to survive within macrophages and establish infection in mammalian hosts. Conversely, if other virulence factors are equally important, TR overexpression alone may have limited effects on infectivity.

Therefore, this study aimed to investigate the hypothesis that the lower activity of TR in *L. tarentolae* contributes to its nonpathogenicity in mammalian hosts and that overexpression of this enzyme can increase infectivity in mammalian cells and tissues. By demonstrating that elevating the activity of a single antioxidant enzyme can significantly increase infectivity, this study provides a unique opportunity to understand the evolution of infectivity-associated traits, the contribution of individual parasite factors to host infection and reinforce TR as a high-priority drug target.

## 2 Materials and methods

### 2.1 Ethical considerations

The Ethics Committee approved the use of the animals in this study under the Use of Animals license from Oswaldo Cruz Institute (CEUA L-002/2022).

### 2.2 Parasites

Mutant and wild-type strains of *L. infantum* (MHOM/MA/67/ITMAP263) and *L. tarentolae* (LV-414) promastigotes were cultured in Schneider’s *Drosophila* medium (Sigma–Aldrich, St. Louis, MO, United States) supplemented with 20% or 10% fetal bovine serum (v/v), 100 U/mL penicillin, and 100 μg/mL streptomycin. The parasites were maintained at 26 °C in a BOD-type incubator. Mutant strains were regularly treated with 50 μg/mL geneticin.

### 2.3 Nucleotide and amino acid sequence alignment

The nucleotide and amino acid sequences of *L. infantum* (LINF_050008500) and *L. tarentolae* (LtaP05.0360) TR were obtained from the TriTrypDB database. Nucleotide and amino acid sequence alignments were performed using Clustal Omega and PROMALS3D, respectively.

### 2.4 Trypanothione reductase overexpression

#### 2.4.1 Polymerase chain reaction

*L. tarentolae* promastigotes were maintained in Schneider’s *Drosophila* medium for 4 days. On the fourth day, the promastigotes were collected and centrifuged at 1000 × g for 10 minutes at 4 °C. DNA was subsequently extracted using a Wizard^®^ Genomic DNA Purification Kit (Promega, Madison, WI, USA) following the manufacturer’s instructions. The extracted DNA was eluted in nuclease-free water and stored at -20 °C until analysis. The DNA concentration and purity were measured with a NanoDrop Lite Plus spectrophotometer (Thermo Fisher Scientific, Waltham, MA, USA). PCR was conducted using a Taq DNA Polymerase Kit (Invitrogen, Carlsbad, CA, USA) with trypanothione reductase-specific primers that contained restriction sites for BamHI (ggatcc) and HindIII (aagctt) or with primers targeting the neomycin resistance gene (**Supplementary Table 1**). The amplification products were examined on a 1% agarose gel using horizontal electrophoresis at 80 V for 90 minutes.

#### 2.4.2 Molecular construction of TR inserts

TR clones containing the BamHI (ggatcc) and HindIII (aagctt) restriction sites were obtained using the PureLink Quick Gel Extraction PCR Purification Kit (Invitrogen). The purified DNA fragments and the pSP72αNEOα vector were separately digested with the restriction enzymes BamHI and HindIII (New England Biolabs, Ipswich, MA, USA) at 37 °C overnight. The digestion products were purified using the PureLink Quick Gel Extraction Kit (Invitrogen) and incubated with T4 DNA ligase (3:1 insert:vector ratio) at 16 °C overnight. After the ligation reaction, either the recombinant vector obtained (LtTRtar + pSP72αNEOα) or the empty vector (pSP72αNEOα) was introduced into competent *E. coli* DH5α cells by heat-shock transformation. After transformation, colonies were selected by plating on LB medium supplemented with 100 µg/mL ampicillin. The recombinant vectors were subsequently purified using a Plasmid Miniprep Purification Kit (Sigma–Aldrich, Saint Louis, MO, USA). The insertion of the TR gene in the pSP72αNEOα vector was confirmed by restriction endonuclease digestion with BamHI and HindIII.

#### 2.4.3 Generation of mutant strains

*Leishmania tarentolae* lines overexpressing TR were generated using the pSP72αNEOα episomal expression vector. This vector replicates autonomously in *Leishmani*a species and does not integrate into the genome. The vector carries a neomycin phosphotransferase gene for antibiotic selection.

Four-day-old wild-type *L. tarentolae* promastigotes were centrifuged, washed, and resuspended in electroporation buffer (21 mM HEPES, 137 mM NaCl) containing five micrograms of the recombinant vector (LtTRtar + pSP72αNEOα) or empty vector (pSP72αNEOα) in cuvettes. Afterward, the parasites were subjected to electroporation at 450 V and 550 µF capacitance for more than 10 seconds, and three parasite lines were generated: (i) LtWT (wild-type *L. tarentolae*), (ii) LtPSP (*L. tarentolae* electroporated with empty vector), and (iii) LtTRtar (*L. tarentolae* with TR overexpression). All the lines were maintained in geneticin (50 µg/mL) for selection to ensure stable episomal vector maintenance and continuous transgene expression. Transfection was later confirmed by PCR using primers specific for the neomycin resistance gene. The stability of TR expression was confirmed by consistent measurements of enzymatic activity across all experiments.

### 2.5 Separation of the soluble fraction and assessment of the enzymatic activity of trypanothione reductase

To obtain the soluble fraction, four-day-old wild-type or mutant *L. infantum* and *L. tarentolae* promastigotes were centrifuged at 1000 × g for 10 minutes. The supernatant was discarded, and the pellet was resuspended in buffer containing a protease inhibitor (40 mM Tris–HEPES (pH 7.5), 1 mM EDTA (pH 8), 250 mM sucrose, 10 mM KCl, 1 mM PMSF, and 10 μM E-64). After resuspension, the cells were lysed by repeated freeze–thaw cycles, consisting of freezing in liquid nitrogen followed by thawing, and then centrifuged at 17,500 × g. The supernatant was considered the soluble fraction.

A Nanodrop spectrophotometer was used to measure the protein concentration. TR activity was assessed using the Ellman reaction (Hamilton et al., 2003). The reaction buffer consisted of 40 mM Tris–HEPES buffer (pH 7.5), 1 mM EDTA, 0.1 mM NADPH, 1 µM oxidized trypanothione (T[S]_2_), and 0.1 mM DTNB. The reaction was initiated by adding the soluble fraction at a final concentration of 0.4 mg/mL. TR activity, indicated by the formation of 2TNB-, was measured colorimetrically at 410 nm (SpectraMax Gemini XPS, Molecular Devices, Silicon Valley, USA) for more than 25 minutes.

### 2.6 Growth curves of wild-type and mutant *L. tarentolae* promastigotes

Wild-type and mutant *L. tarentolae* promastigotes were cultured for 4 days. On the fourth day, the parasites were incubated in Schneider’s *Drosophila* medium (Sigma–Aldrich, Saint Louis, MO, USA) at an initial concentration of 1 × 10^6^ parasites/mL. Promastigotes were counted in a Neubauer chamber daily for 6 days.

### 2.7 Evaluation of hydrogen peroxide tolerance in wild-type and mutant *L. tarentolae*

*L. infantum*, wild-type and TR-overexpressing *L. tarentolae* promastigotes were cultured in medium for 4 days. Parasites were then adjusted to a density of 1 × 10^6^ parasites/mL and exposed to increasing concentrations of freshly prepared H_2_O_2_ (125–1000 µM). After 72 hours, viable parasite recovery after oxidative challenge was evaluated using the resazurin reduction assay, based on the conversion of resazurin to fluorescent resorufin by viable cells. Fluorescence was measured using a SpectraMax Gemini XPS microplate reader (Molecular Devices, Silicon Valley, USA), with excitation at 560 nm and emission at 590 nm.

### 2.8 Evaluation of the infectivity of wild-type and mutant *L. tarentolae* promastigotes

Murine peritoneal macrophages from BALB/c mice were collected in RPMI medium (Sigma–Aldrich, Saint Louis, MO, USA) supplemented with 10% fetal bovine serum, 1% glutamine, and 1% pyruvate. A total of 1×10^6^ macrophages/mL were plated in the wells of Lab-Tek chamber slides (Thermo Fisher Scientific, Waltham, MA, USA) and incubated at 37 °C with 5% CO_2_ for 1 hour. Nonadherent macrophages were removed by washing with RPMI. Adherent macrophages were infected with wild-type, empty-vector control, or TR-overexpressing *L. tarentolae* promastigotes and *L. infantum* promastigotes at a ratio of 3 parasites per macrophage (MOI 3:1) for 5 hours at 37 °C with 5% CO_2_. After 5 hours, nonadherent parasites were removed by gentle washing, and infected macrophages were maintained in culture for 24 or 72 hours. The slides were stained with the hematological stain InstantProv (Newprov, Curitiba, Brazil), and the infection index was calculated as follows: Infection index = (% infected macrophages) × (average number of amastigotes per infected macrophage). For each condition, at least 200 macrophages were counted per slide across randomly selected microscopic fields under a light microscope.

### 2.9 Evaluation of the role of trypanothione reductase in the infectivity of *L. tarentolae in vivo*

To evaluate the role of trypanothione reductase in infectivity *in vivo*, female BALB/c mice (5 animals per group) maintained under pathogen-free conditions were intraperitoneally infected with wild-type or mutant *L. tarentolae* promastigotes (1x10^8^ promastigotes/10 µL of PBS). The mice were euthanized 7 days after infection for liver removal and analysis of the parasite load.

#### 2.9.1 Quantification of the parasite load

Seven days after infection, mice infected with wild-type or mutant *L. tarentolae* promastigotes were euthanized to analyze the parasite load by real-time PCR. The liver of each mouse was removed, weighed, and macerated in Schneider’s *Drosophila* medium supplemented with 10% fetal bovine serum. DNA was extracted from fifty milligrams of homogenized using the phenol–chloroform method. The DNA concentration and purity were measured using a Nanodrop One spectrophotometer (Thermo Fisher Scientific, Waltham, MA, USA), and real-time PCR was conducted with a GoTaq^®^ qPCR Master Mix Kit, 800 ng of total DNA, and primers specific to ssrRNA (Prina et al., 2007).

### 2.10 Statistical analysis

The data were analyzed using GraphPad Prism 7 (GraphPad Software, La Jolla, CA, USA). Comparisons between two groups were performed using Student’s t test. Comparisons among three or more groups were performed using one-way ANOVA followed by Tukey’s *post hoc* test. Statistical significance was set at p ≤ 0.05. All data are presented as the mean ± standard error (SE) of at least three independent experiments.

## 3 Results

### 3.1 *Leishmania infantum* exhibits markedly higher TR Activity than *L. tarentolae*

**Figure 1:**
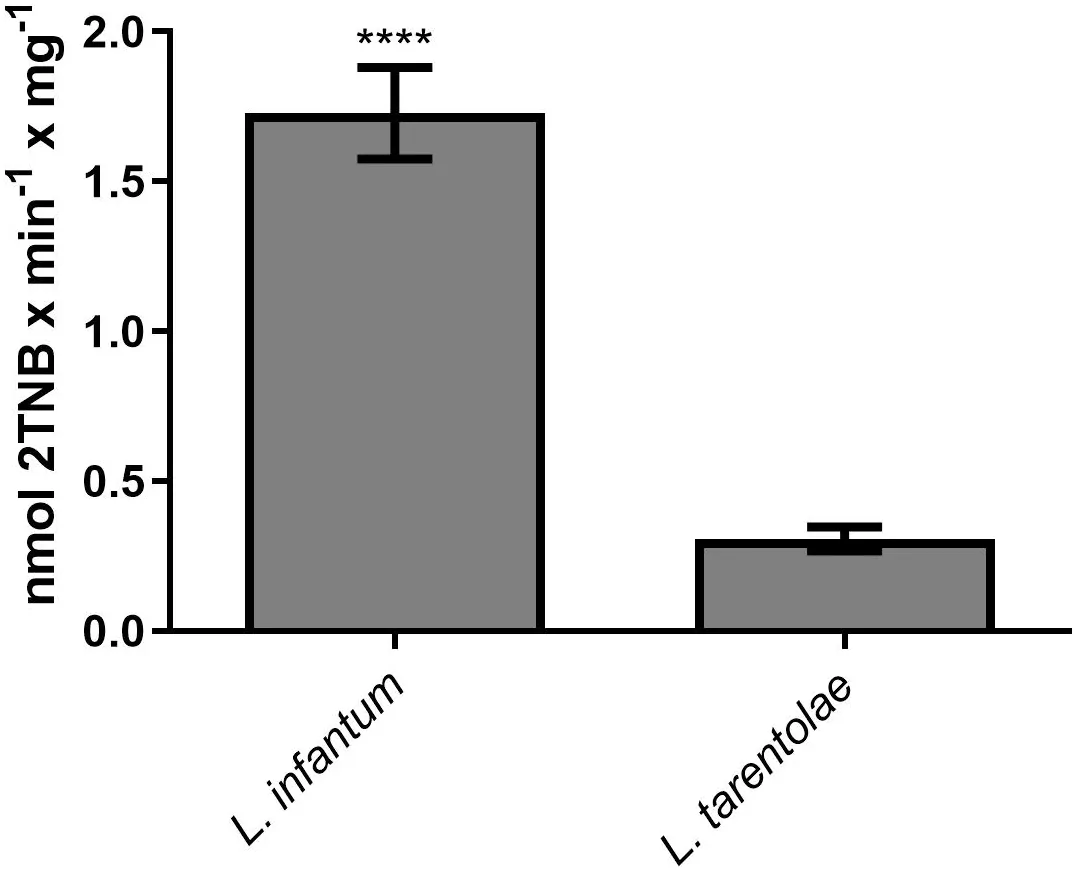
Baseline comparison of TR activity between *L. infantum* and *L. tarentolae*. TR activity in the soluble fraction of *L. infantum* and *L. tarentolae* promastigotes was measured using the Ellman reaction. The data are presented as the mean ± standard error of the 2TNB formation rate (nmol × min^-1^ × mg^-1^) from 3 independent experiments, each with 3 technical replicates. Statistical analysis was performed using Student’s t test. **** p < 0.0001.

To establish an experimental baseline for the role of TR in infectivity-associated phenotypes, we first compare the TR enzymatic activity between a known pathogenic species, *L. infantum*, and the nonpathogenic species *L. tar*entolae. This comparison was not intended to represent the diversity of all pathogenic and nonpathogenic *Leishmania* species, but rather to define the baseline difference between the two species used in the present study. The activity of TR from pathogenic *Leishmania infantum* was greater than that of TR from nonpathogenic *Leishmania tarentolae* (**Figure 1**). Specifically, the activity of TR from *L. infantum* was 5.7 times greater than that of TR from *L. tarentolae* (1.72 nmol 2TNB x min^-1^ x mg^-1^ and 0.3 nmol 2TNB x min^-1^ x mg^-1^, respectively). Thus, these results show that *L. infantum* exhibited higher TR activity than *L. tarentolae*, supporting investigation into the molecular basis and functional consequences of this difference.

### 3.2 Critical amino acid substitutions in the *L. tarentolae* TR protein explain its reduced enzymatic activity

**Figure 2:**
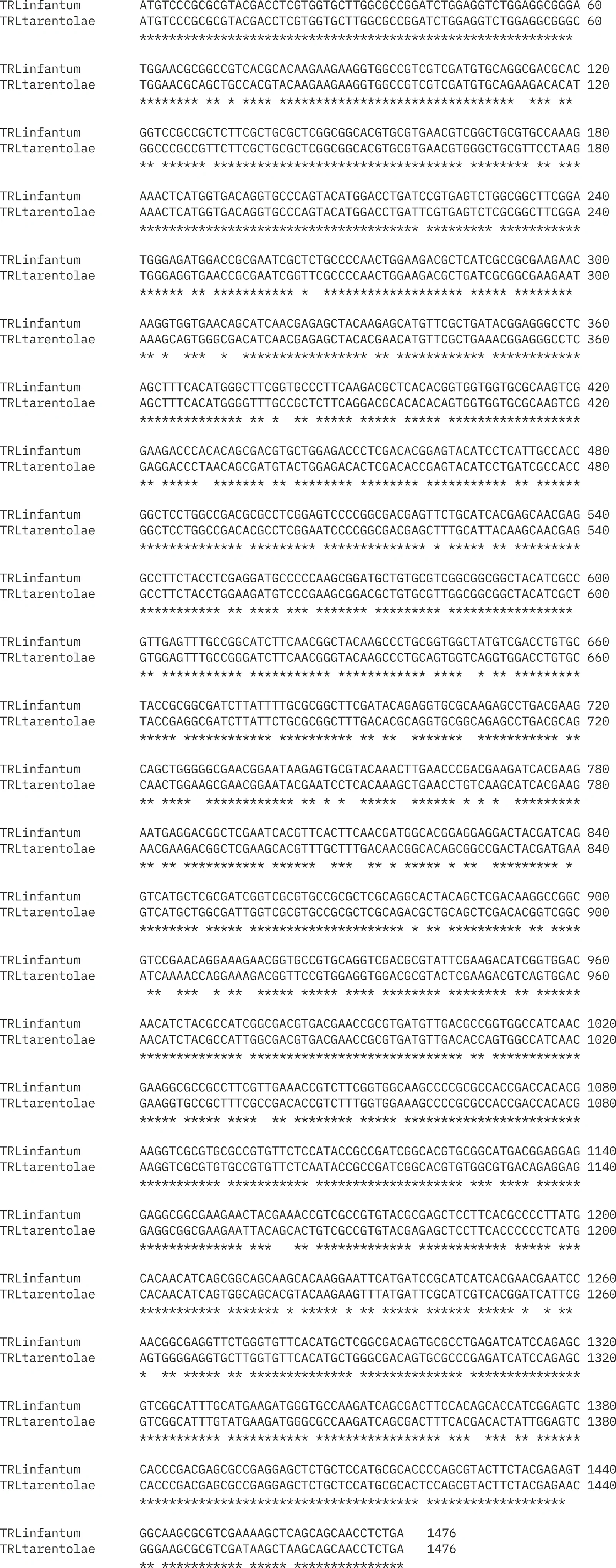
Nucleotide sequence alignment of the TR genes from *L. infantum* and *L. tarentolae*. Alignment was performed using Clustal Omega. The asterisks (*) indicate the positions of nucleotide identity between the two sequences. TRLtarentolae: trypanothione reductase sequence from *L. tarentolae* (TriTrypDB: LtaP05.0360). TRLinfantum: trypanothione reductase sequence from *L. infantum* (TriTrypDB: LINF_050008500).

Having observed a significant difference in TR activity, we next sought to identify the genetic basis of this difference by comparing the nucleotide sequences of the TR genes from both species. The TR sequences for both *Leishmania* species were retrieved from the TriTrypDB database (*L. infantum*: LINF_050008500; *L. tarentolae*: LtaP05.0360), and the sequences were aligned using Clustal. The nucleotide sequences of TR from the two parasites were 87.26% similar (**Figure 2**). Although substantial similarity was observed at the nucleotide level, nucleotide sequences are insufficient to explain functional differences, as variations in nucleotide composition do not necessarily correspond to changes in amino acid sequences or protein structures. Consequently, an investigation into the resulting amino acid sequences was conducted.

### 3.3 Structural differences in the TR catalytic site and substrate recognition domain correlate with reduced activity

**Figure 3:**
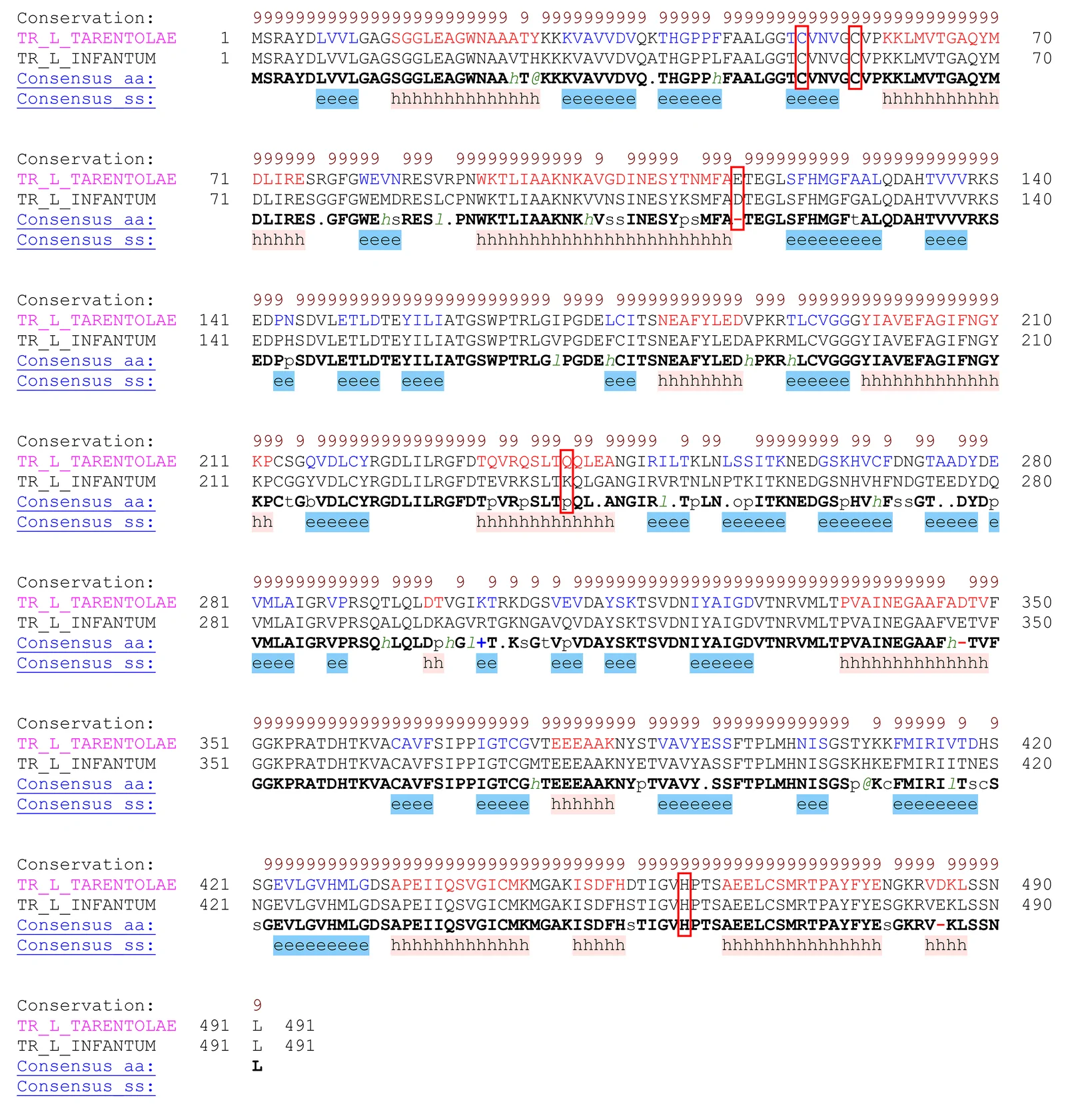
The TR proteins from *L. infantum* and *L. tarentolae* share 87% amino acid sequence identity but differ at key catalytic residues. Amino acid sequence alignment of trypanothione reductases from *L. infantum* and *L. tarentolae*. Cysteine 52, cysteine 57, and histidine 461, which constitute the catalytic triad, are highlighted by the red rectangles. Secondary structure symbols: h, alpha helix; e, beta sheet. The number 9 indicates the number of identical residues at that position. TR_L_TARENTOLAE: trypanothione reductase sequence from *L. tarentolae* (TriTrypDB: LtaP05.0360). TR_L_INFANTUM: trypanothione reductase sequence from *L. infantum* (TriTrypDB: LINF_050008500).

To understand how genetic differences translate to function, we analyzed the primary and secondary protein structures of the TR enzyme, focusing on amino acid substitutions in critical domains. The primary structure of a protein determines its folding into a three-dimensional shape, which in turn influences its activity. In this context, alignment of the TR amino acid sequences from *L. infantum* (LINF_050008500) and *L. tarentolae* (LtaP05.0360) was performed using the PROMALS3D web server. The alignment revealed 87% sequence similarity (**Figure 3**). The amino acids Cys 52, Cys 57, and His 461, which form the enzyme’s catalytic site, are highlighted in the sequences and were found to be highly conserved across species. The amino acids Asp 116 and Lys 240, which are involved in substrate recognition by TR, are also highlighted, but they exhibited critical differences. Both Asp 116 and Lys 240 were shown to be present in TR from *L. infantum* but are absent in TR from *L. tarentolae*, with Asp 116 being replaced by Glu and Lys 240 is replaced by Gln an amino acid from a different family. Except for these polymorphisms, no significant differences were found in the predicted protein secondary structures of TR from the two species. Collectively, these structural analyses suggest that specific amino acid substitutions, particularly at the substrate recognition site, could be a primary factor contributing to the reduced enzymatic activity observed in *L. tarentolae* TR.

### 3.4 Overexpression of TR increases its enzymatic activity by 6-fold in *L. tarentolae* promastigotes

**Figure 4:**
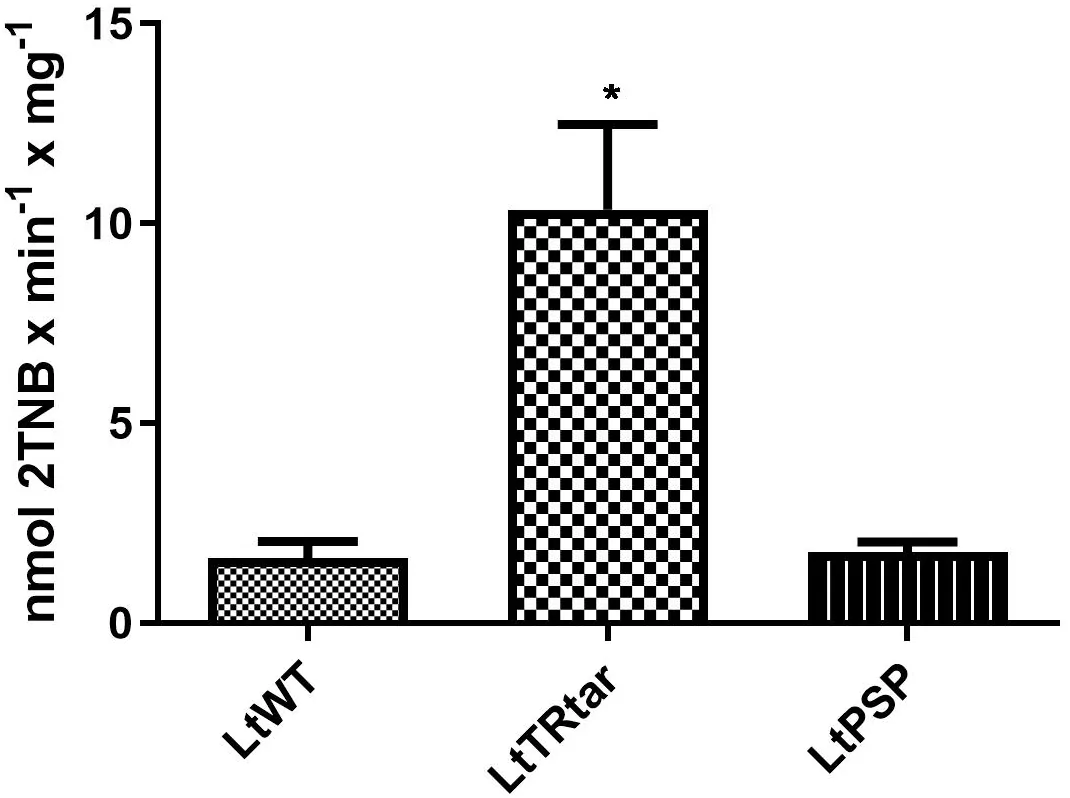
TR overexpression results in a 6-fold increase in enzymatic activity in L. tarentolae promastigotes. TR activity in soluble fractions obtained from wild-type and transfected parasites was measured using the Ellman method. The data are presented as the mean ± standard error of the 2TNB formation rate (nmol × min^-1^ × mg^-1^) from 3 independent experiments, each with 2 technical replicates. Statistical analysis was performed using one-way ANOVA followed by Tukey’s *post hoc* test. * p < 0.05. LtWT = wild-type *L. tarentolae* promastigotes; LtTRtar = *L. tarentolae* promastigotes transfected with the recombinant vector pSP72αNEOα + TR of *L. tarentolae*; LtPSP = *L. tarentolae* promastigotes transfected with the empty vector pSP72αNEOα.

To directly test the hypothesis that TR activity contributes to parasite infectivity, our next objective was to create a gain-of-function model by overexpressing TR in nonpathogenic *L. tarentolae* and confirming the resulting increase in enzymatic activity. A recombinant vector containing the TR gene (pSP72αNEOα + TRtar) and an empty vector (pSP72αNEOα) were constructed and transfected into *L. tarentolae* promastigotes. The construction of the recombinant vector was confirmed by restriction analysis with BamHI and HindIII (**Supplementary Figure 1**), and successful transfection was confirmed by PCR using primers specific for the neomycin selection marker (**Supplementary Figure 2**). The overexpression of TR in *L. tarentolae* promastigotes (WT and mutant) was subsequently confirmed by measuring TR activity using the Ellman method (**Supplementary Figure 3**). The TR enzymatic activity of the LtTRtar strain was 6-fold greater than that of the wild-type (LtWT) or empty-vector control (LtPSP) strains (1.63 nmol 2TNB x min^-1^ x mg^-1^; 10.34 nmol 2TNB x min^-1^ x mg^-1^ and 1.76 nmol 2TNB x min^-1^ x mg^-1^, for LtWT, LtTRtar and LtPSP, respectively; p < 0.05) (**Figure 4**). These results validate our experimental model and confirm that the engineered parasites exhibited significantly enhanced TR activity, thereby enabling subsequent functional assays.

### 3.5 TR overexpression increases parasite growth and proliferation

**Figure 5:**
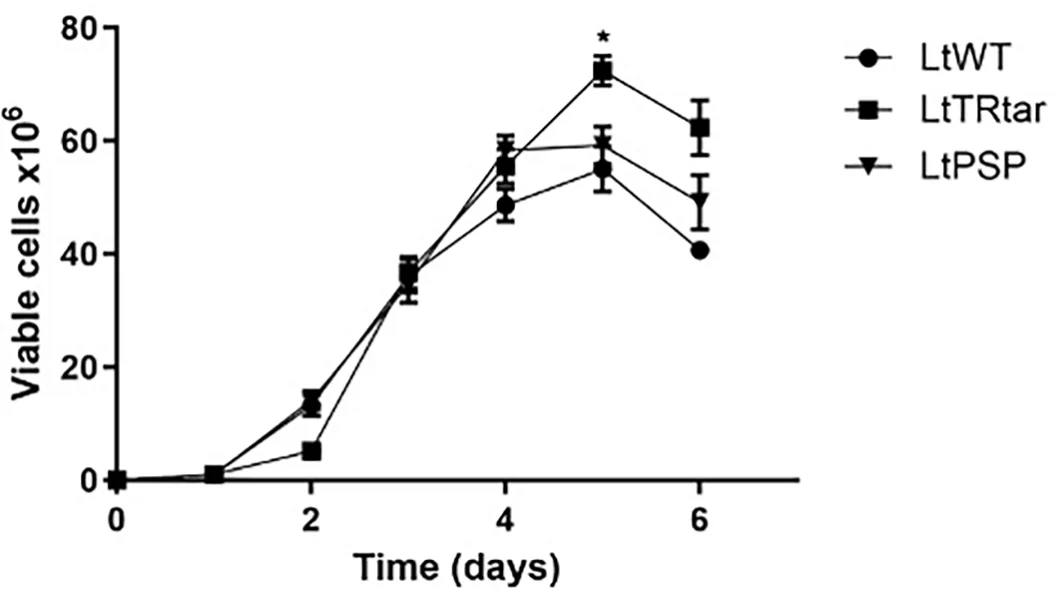
Compared with wild-type parasites, TR-overexpressing *L. tarentolae* promastigotes exhibit accelerated growth. Growth was initiated by inoculating 1×10^6^ parasites/mL into a total volume of 10 mL of Schneider’s Drosophila medium. Parasites were counted over a 6-day period using a Neubauer chamber. The data are presented as the mean ± standard error from 3 independent experiments, each with 3 technical replicates. Statistical analysis was performed using one-way ANOVA followed by Tukey’s *post hoc* test. * p < 0.05. LtWT = wild-type *L. tarentolae* promastigotes; LtTRtar = *L. tarentolae* promastigotes transfected with the recombinant vector pSP72αNEOα + TR of *L. tarentolae*; LtPSP = *L. tarentolae* promastigotes transfected with the empty vector pSP72αNEOα.

Following the confirmation of increased TR activity, we evaluated the effects of this overexpression on fundamental parasite biology, specifically growth and proliferation. WT and mutant *L. tarentolae* promastigotes were cultured in Schneider’s Drosophila medium at an initial concentration of 1 × 10^6^ parasites/mL, and promastigotes were counted daily for 6 days. The proliferation profiles of the parasites varied slightly. On the fifth day of culture, compared with wild-type and pSP72αNEOα-transfected parasites, *L. tarentolae* parasites overexpressing TR exhibited greater proliferation (p = 0.034; **Figure 5**). This finding indicates that TR activity is not only linked to stress resistance but also promotes the growth of *L. tarentolae* parasites under standard culture conditions.

### 3.6 TR overexpression confers increased resistance to oxidative stress

**Figure 6:**
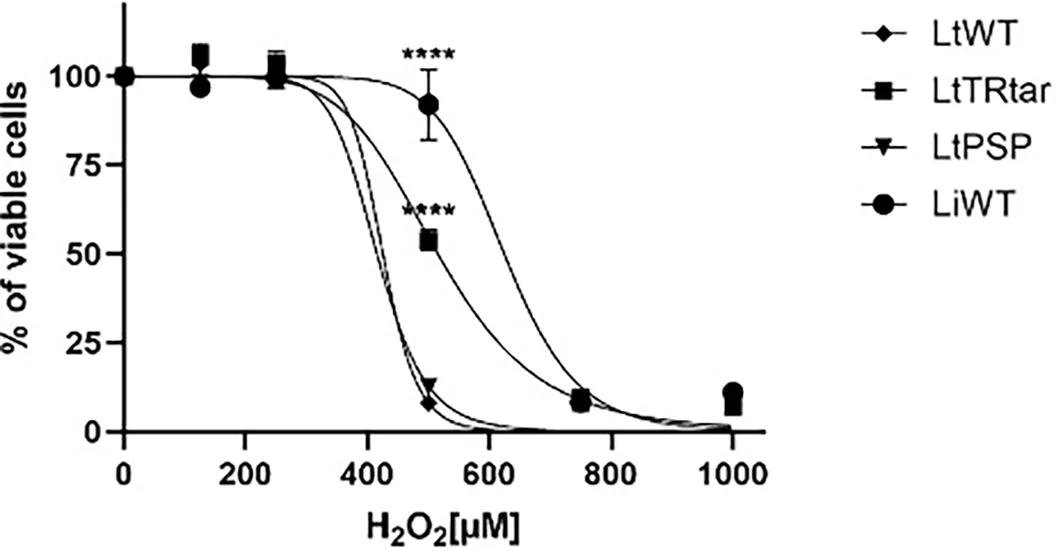
TR overexpression enhances tolerance of *L. tarentolae* promastigotes to hydrogen peroxide-induced oxidative stress. Promastigotes (1×10^6^ parasites/mL) were incubated in the absence or presence of H_2_O_2_ (125–1000 µM) for 72 hours. Cell viability was assessed using a resazurin reduction assay and measured by fluorescence. The data are presented as the mean ± standard error from 3 independent experiments, each with 3 technical replicates. Statistical analysis was performed using one-way ANOVA followed by Tukey’s *post hoc* test. **** p < 0.001. LtWT = wild-type *L. tarentolae* promastigotes; LtTRtar = *L. tarentolae* promastigotes transfected with the recombinant vector pSP72αNEOα + TR of *L. tarentolae*; LtPSP = *L. tarentolae* promastigotes transfected with the empty vector pSP72αNEOα; LiWT = wild-type *L. infantum* promastigotes.

To determine whether increased TR activity translates to a more robust antioxidant defense, we next assessed the resistance of the parasites to direct oxidative challenge. Wild-type and mutant *L. tarentolae* promastigotes were exposed to increasing concentrations of hydrogen peroxide for 72 hours. Compared with wild-type and pSP72αNEOα-transfected *L. tarentolae* parasites, which had IC_50_ values for hydrogen peroxide of 425 µM and 415 µM, respectively, *L. tarentolae* parasites overexpressing the trypanothione reductase gene exhibited lower sensitivity to hydrogen peroxide, with an IC_50_ of 515 µM (**Figure 6**). These findings indicate that the observed 6-fold increase in TR enzymatic activity increased the ability of *L. tarentolae* promastigotes to maintain viability after exogenous oxidative challenge, a key stress condition encountered within host macrophages.

### 3.7 TR overexpression dramatically increases macrophage infectivity

**Figure 7:**
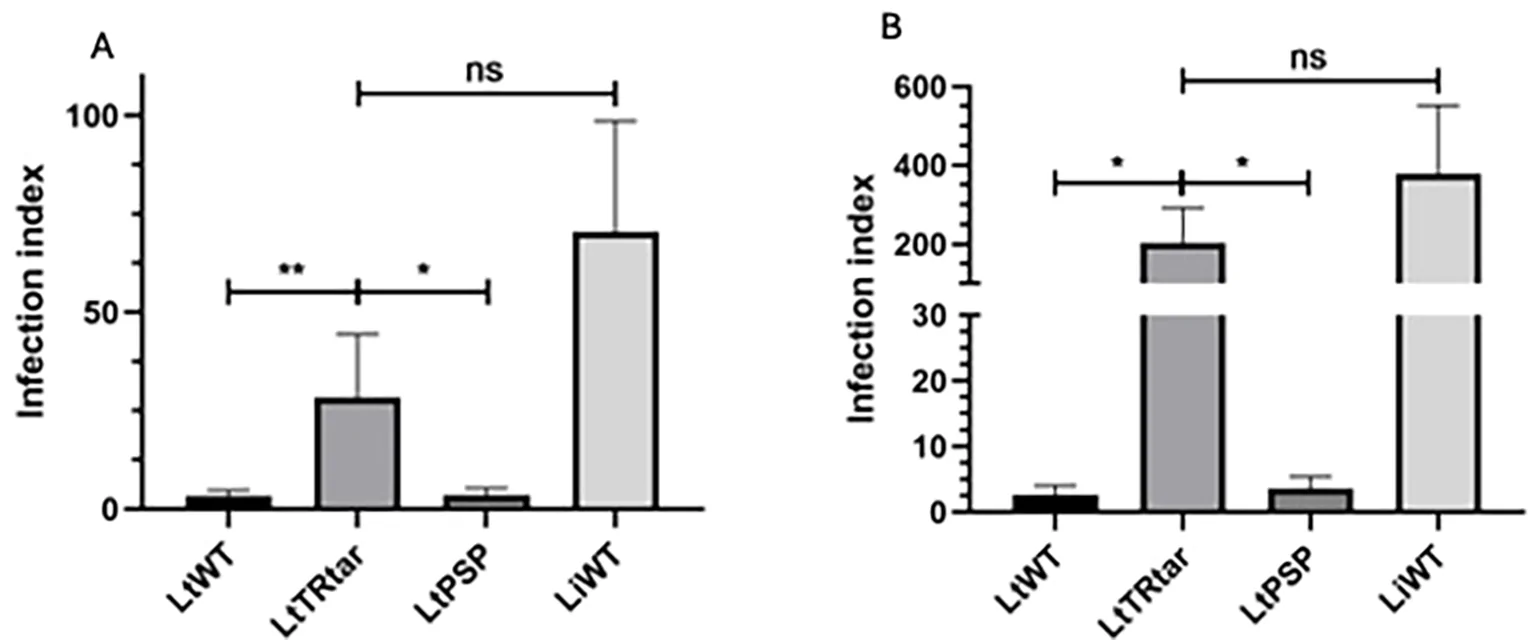
TR overexpression dramatically increases *L. tarentolae* infectivity in murine peritoneal macrophages, with the effect amplifying over time. Murine peritoneal macrophages were infected with wild-type *L. tarentolae* and *L. infantum* promastigotes and mutant *L. tarentolae* promastigotes for 5 hours at a ratio of 3 parasites per macrophage. The interaction between parasites and macrophages was maintained for 24 hours **(A)** or 72 hours **(B)** in an incubator at 37 °C with 5% CO_2_. The data are presented as the mean ± standard error from 3 independent experiments, each with 2 technical replicates. Statistical analysis was performed using one-way ANOVA followed by Tukey’s *post hoc* test. * p < 0.05; ** p < 0.01. LtWT = wild-type *L. tarentolae* promastigotes; LtTRtar = *L. tarentolae* promastigotes transfected with the recombinant vector pSP72αNEOα + TR of *L. tarentolae*; LtPSP = *L. tarentolae* promastigotes transfected with the empty vector pSP72αNEOα; LiWT = wild-type *L. infantum* promastigotes.

To determine whether elevated resistance to oxidative stress increases parasite infectivity, we evaluated the role of TR in infectivity *in vitro*. Wild-type *L. infantum* and both wild-type and mutant *L. tarentolae* promastigotes were incubated with murine peritoneal macrophages for 24 and 72 hours, after which 200 macrophages per sample were counted by light microscopy to determine the infection index. A substantially increased infection index for *L. tarentolae* parasites overexpressing TR was observed at both time points. At 24 hours, the infection index of these parasites was 8-fold greater than that of wild-type or pSP72αNEOα-transfected parasites (**Figure 7A**). The effect was even more pronounced at 72 hours, when the infection index of the TR-overexpressing parasites was approximately 78- and 56-fold higher than those of the wild-type and pSP72αNEOα-transfected parasites, respectively (**Figure 7B**). Notably, no significant difference in the infection index was detected between *L. tarentolae* parasites overexpressing the TR gene and wild-type *L. infantum* parasites or between wild-type *L. tarentolae* and pSP72αNEOα-transfected *L. tarentolae* parasites. These results provide strong evidence that elevated TR activity is associated with a marked increase in the ability of nonpathogenic *L. tarentolae* to infect and persist within mammalian macrophages.

### 3.8 TR overexpression increases parasite survival and liver colonization in mice

**Figure 8:**
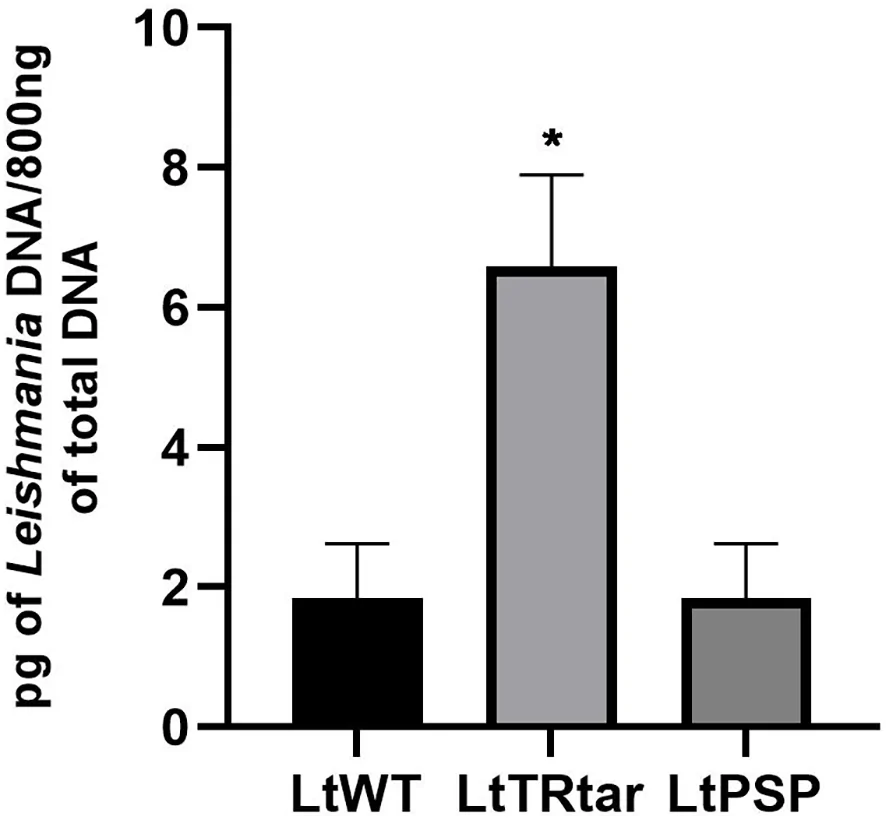
TR-overexpressing *L. tarentolae* parasites establish significantly higher liver parasite loads in BALB/c mice. BALB/c mice were infected with wild-type and mutant *L. tarentolae* promastigotes and euthanized 7 days after infection. The liver was removed, and total DNA was extracted. The parasite load was quantified by real-time PCR using primers specific to *Leishmania* ssrRNA (800 ng of total DNA per sample). The data are presented as the mean *Leishmania* DNA mass (in nanograms) per 800 ng of total DNA ± standard error (5 animals per group, with 3 technical replicates per experiment). Statistical analysis was performed using one-way ANOVA followed by Tukey’s *post hoc* test. * p < 0.05. LtWT = wild-type *L. tarentolae* promastigotes; LtTRtar = *L. tarentolae* promastigotes transfected with the recombinant vector pSP72αNEOα + *L. tarentolae* TR; LtPSP = *L. tarentolae* promastigotes transfected with the empty vector pSP72αNEOα.

Finally, to assess whether the observed *in vitro* effects on infectivity translate into an increased parasite persistence *in vivo*, we used a murine infection model. Female BALB/c mice were infected intraperitoneally with wild-type or mutant *L. tarentolae* promastigotes and euthanized 7 days after infection to measure the parasite load in the liver. Compared with mice infected with wild-type parasites or parasites transfected with the pSP72αNEOα vector, mice infected with *L. tarentolae* promastigotes overexpressing TR presented significantly increased liver parasite loads (increases of 3.3- and 3.7-fold, respectively) (**Figure 8**). These results confirm that the overexpression of TR in *L. tarentolae* parasites increases infectivity *in vivo*, supporting a functional link between the TR activity level and the ability of a parasite to infect the mammalian host.

## 4 Discussion

This study aimed to investigate the hypothesis that TR activity is a key determinant of parasite infectivity and oxidative-stress tolerance. Our findings provide compelling evidence that TR is an important infectivity-associated factor in *Leishmania* parasites. By overexpressing TR in the nonpathogenic species *L. tarentolae*, we increased its resistance to oxidative stress, resulting in a dramatic increase in infectivity in both macrophages and a murine infection model. These findings suggest that TR-dependent redox homeostasis is an important contributor to infection-associated phenotypes in *Leishmania*. However, because virulence is a multifactorial trait, our data should not be interpreted as evidence that TR activity alone is sufficient to confer full virulence.

During their life cycles, parasites in the *Leishmania* clade are exposed to various reactive oxygen species (ROS), such as superoxide anions, hydroxyl radicals, and hydrogen peroxide (H_2_O_2_) (Santi and Murta, 2022). The highly oxidative environment they encounter can damage a range of cellular components, including lipids, proteins, nucleic acids, and carbohydrates (Reverte et al., 2022). In the vertebrate host, ROS production is a primary immune response against *Leishmania* infection, and mammal-infecting parasites must maintain effective redox homeostasis to withstand these oxidative bursts (Krauth-Siegel et al., 2003). The genome of *L. tarentolae* has been sequenced, revealing an underrepresentation of genes encoding proteins involved in redox homeostasis (Raymond et al., 2012).

Our findings revealed lower TR activity in wild-type *L. tarentolae* than in *L. infantum*, suggesting that the ability of *L. tarentolae* parasites to protect against oxidative stress is inherently limited. The role of TR in parasite survival, infectivity, and pathogenicity-related processes has been demonstrated in the literature (Dumas et al., 1997; Krieger et al., 2000; González-Chávez et al., 2019). Additionally, *L. tarentolae* has been reported to lack genes involved in oxidative stress resistance (Raymond et al., 2012).

The catalytic efficiency of an enzyme is directly determined by its three-dimensional structure and the chemical properties of its active site. Since we observed a striking difference in enzymatic activity between TR proteins from *L. infantum* and *L. tarentolae*, we examined potential differences in their primary and secondary structures. Alignments of nucleotide and amino acid sequences revealed variations in residues essential for catalysis and substrate recognition. Key residues involved in substrate binding and catalytic turnover differ between the two *Leishmania* species. Notably, Asp116 and Lys240, which are important for substrate recognition, are present in *L. infantum* but are substituted by other amino acids in *L. tarentolae*. Asp116 aids in the recognition of the T(S)_2_ substrate by TR in *Trypanosoma congolense* (Henderson et al., 1991).

Furthermore, Lys240 in the catalytic cleft of *L. infantum* TR forms an electrostatic interaction with trypanothione, facilitating its transport to the cysteine residues at the catalytic site (Baiocco et al., 2013). The substitution of Lys240 with Gln in *L. tarentolae* TR, together with the substitution of Asp116 with Glu and other amino acid differences identified in our alignment, may contribute to the lower TR activity observed in soluble fractions of *L. tarentolae*. However, additional biochemical studies would be required to confirm their direct effect on enzyme-specific activity.

Building on this structural understanding, we found that TR activity is directly linked to parasite growth and proliferation. Krieger and colleagues linked TR levels in knockout-induced *T. brucei* cells to parasite growth: a TR activity level less than 10% of the wild-type level impaired growth, whereas restoring TR activity enabled the parasites to proliferate (Krieger et al., 2000). This evidence supports the role of TR in growth dynamics, as shown by our observation that the growth rate of TR-overexpressing *L. tarentolae* promastigotes was greater than that of wild-type promastigotes and promastigotes transfected with the empty plasmid. These findings link TR not only to survival under stress but also to parasite replication.

The increased resistance to oxidative stress conferred by TR overexpression may contribute to the increased infectivity observed in macrophages. A decrease in TR activity in *T. brucei* has been shown to reduce its ability to infect mice compared with that of wild-type *T. brucei* (Krieger et al., 2000). The trypanothione system functions as an integrated antioxidant defense network, with TR serving as the central hub. TR works in concert with other trypanothione-dependent enzymes, particularly tryparedoxin peroxidase (TryP), which catalyze the reduction of hydroperoxides using trypanothione as the electron donor (Iyer et al., 2008; Roy et al., 2025). Our results show that TR overexpression in *L. tarentolae* increases tolerance to hydrogen peroxide challenge and is associated with increased infection of murine peritoneal macrophages over 24 and 72 hours, reinforcing the hypothesis that TR-dependent antioxidant capacity contributes to infection-associated phenotypes.

The dramatic increase in infectivity observed in TR-overexpressing *L. tarentolae* promastigotes likely reflects a combination of interconnected effects on host–pathogen interactions within macrophages. Increased TR activity enables the parasite to more efficiently neutralize reactive oxygen species generated by macrophage NADPH oxidase, thereby reducing oxidative stress, preserving cellular integrity, and maintaining a more reduced intracellular environment, preserving the structural and functional integrity of other parasite virulence factors (Roy et al., 2025).

From an evolutionary perspective, the reduced TR activity in *L. tarentolae* reflects the adaptation of this species to fundamentally different host environments. As a reptilian parasite, *L. tarentolae* likely encounters a less challenging oxidative environment (Mendoza-Roldan et al., 2022). Reptilian hosts are thought to generate lower levels of reactive oxygen species in response to parasitic infection than mammalian macrophages, which have evolved specialized mechanisms for intracellular pathogen killing through a potent oxidative burst (Van Assche et al., 2011).

The critical role of TR in surviving this oxidative stress in mammalian hosts has been well documented; *Leishmania* mutants with reduced TR activity show markedly decreased survival in macrophages (Dumas et al., 1997), and *Trypanosoma* parasites lacking TR are entirely avirulent (Krieger et al., 2000). Consequently, *L. tarentolae* may have experienced reduced selective pressure to maintain a highly efficient antioxidant system, a hypothesis supported by genomic data showing an underrepresentation of antioxidant defense genes in this species (Raymond et al., 2012). However, our demonstration that restoring TR activity to the level observed in *L. infantum*, a mammalian pathogen, increases infectivity in mammalian macrophages is a novel contribution of this study, suggesting that the genetic and biochemical machinery required for maintaining effective redox homeostasis is preserved in *L. tarentolae* but remains inactive due to the absence of a challenging oxidative environment.

Compared with wild-type parasites, TR-overexpressing *L. tarentolae* parasites demonstrated markedly increased infectivity in BALB/c mice, resulting in a 3.3-fold higher liver parasite load than wild-type parasites and a 3.7-fold higher liver parasite load than control parasites transfected with an empty vector. Although TR overexpression increased macrophage infectivity and liver parasite burden in mice, disease-associated parameters, including clinical signs, tissue pathology, morbidity, mortality, and long-term persistence, were not evaluated. Therefore, these data should be interpreted as evidence that TR contributes to parasite infectivity, but not as proof that TR overexpression alone causes disease or confers full virulence in mice. This interpretation is further supported by the fact that *L. tarentolae* parasites lack other specific virulence factors present in naturally pathogenic species, such as delta-amastin, lipophosphoglycan (LPG), and acid phosphatase, which are important for immune evasion and intracellular survival in mammalian hosts (Raymond et al., 2012). Consequently, even TR-overexpressing *L. tarentolae* parasites remain vulnerable to certain host defense mechanisms, particularly complement-mediated lysis, which may limit their ability to establish persistent infections in immunocompetent hosts. This reinforces the idea that TR is one of several factors that contributes to the infection-associated phenotype of *Leishmania* parasites, but is not sufficient by itself to confer full pathogenicity.

In addition to providing fundamental insights into parasite infectivity, redox adaptation, and host adaptation, trypanothione reductase has important implications for the development of therapies for leishmaniasis. Epigallocathechin-O-3-gallate (EGCG), a polyphenolic compound in green tea, inhibits TR activity in *L. infantum*, resulting in decreased infectivity both *in vitro* in macrophages and *in vivo* in murine infection models. Similarly, silibinin, a flavonolignan from milk thistle, inhibits TR enzymatic activity and significantly reduces the infection index in *L. infantum*-infected macrophages (Inacio et al., 2019, 2021; Debize da Motta et al., 2025). The finding that modulating TR activity has a dramatic effect on infectivity strongly reinforces TR as a high-priority drug target.

In conclusion, this study provides evidence that trypanothione reductase contributes to *Leishmania* infectivity, but TR overexpression alone is not sufficient to confer full virulence. By demonstrating that TR overexpression increases infection-associated phenotypes, our findings support the concept that redox homeostasis is one important component of the multifactorial network underlying *Leishmania* host adaptation and pathogenic potential. Further studies should investigate whether the simultaneous overexpression of TR and other virulence factors (such as LPG, delta-amastin, acid phosphatase, or gp63) can fully reproduce the infectivity profile of naturally pathogenic species, leading to a deeper understanding of the multifactorial nature of parasite virulence and contributing to the development of novel, multitarget therapeutic strategies to combat leishmaniasis.

## Data Availability

The original contributions presented in the study are included in the article/**Supplementary Material**. Further inquiries can be directed to the corresponding author.
