# Supplementary material for: Trypanothione reductase overexpression increases the infectivity of Leishmania tarentolae through improved redox homeostasis and increased resistance to oxidative stress
Source: Front Cell Infect Microbiol. 2026 Aug 17;16:1824711. doi: 10.3389/fcimb.2026.1824711 (PMC13526550; doi:10.3389/fcimb.2026.1824711)
Supplement: Supplementary file 1 [file DataSheet1.pdf]

Supplementary Table 1. Sequences of the primers used for the polymerase chain reaction. The restriction sites for BamHI and HindIII are underlined in the primer sequences used to overexpress trypanothione reductase.

**Primer**	**Sequence**	
	**Forward**	**Reverse**
Trypanothione reductase-*Bam*HI	CCGGATCCATGTCCCGCG CGT ACGACCT	
Trypanothione reductase-*Hind*III		CCAAGCTTTCAGAGGTTGCT GCTG AGCT
Neomycin	ATGATTGAACAAGATGG ATTG	TCAGAAGAACTCGTCAAGA AG

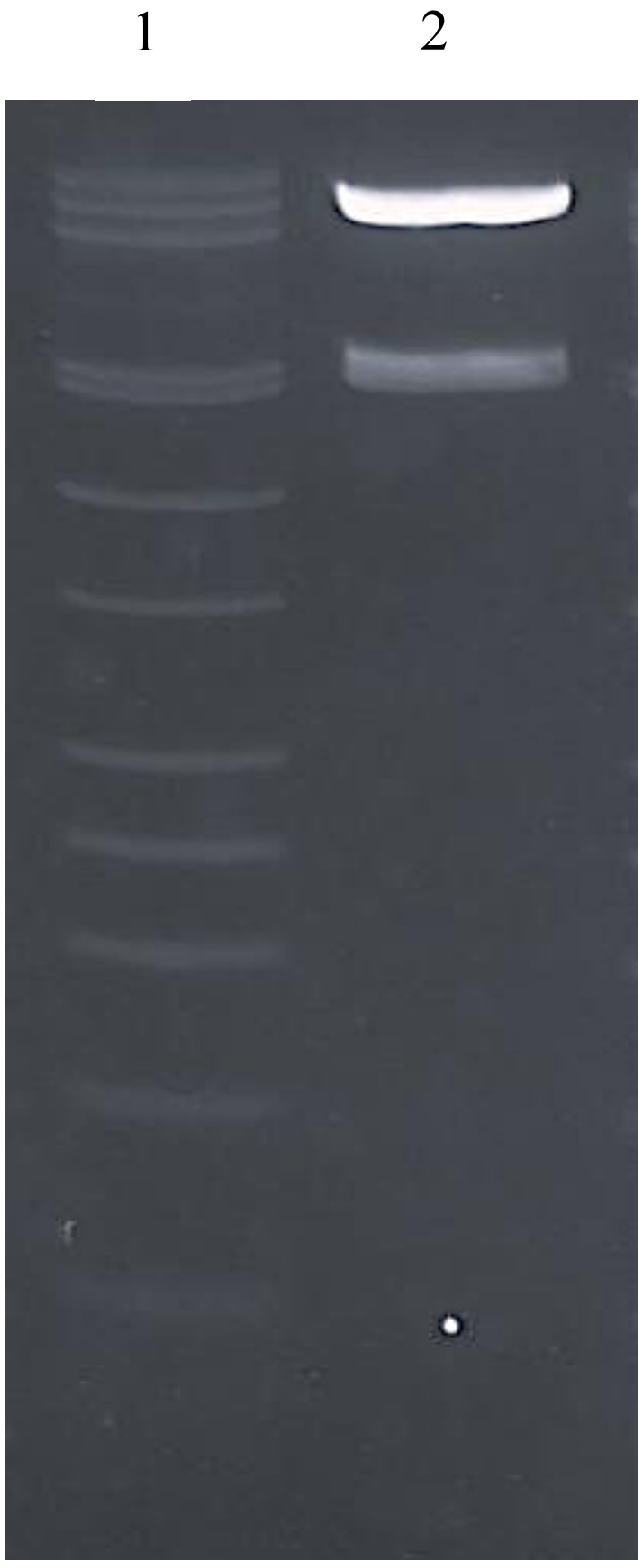

Supplementary Figure 1. Agarose gel electrophoresis (1.5%) results showing the fragments from the digestion of the recombinant vector. Lane 1: Molecular weight marker (50-10000 bp); Lane 2: Products resulting from enzymatic digestion of the recombinant vector pSP72αNEOα + TR of *L. tarentolae*.

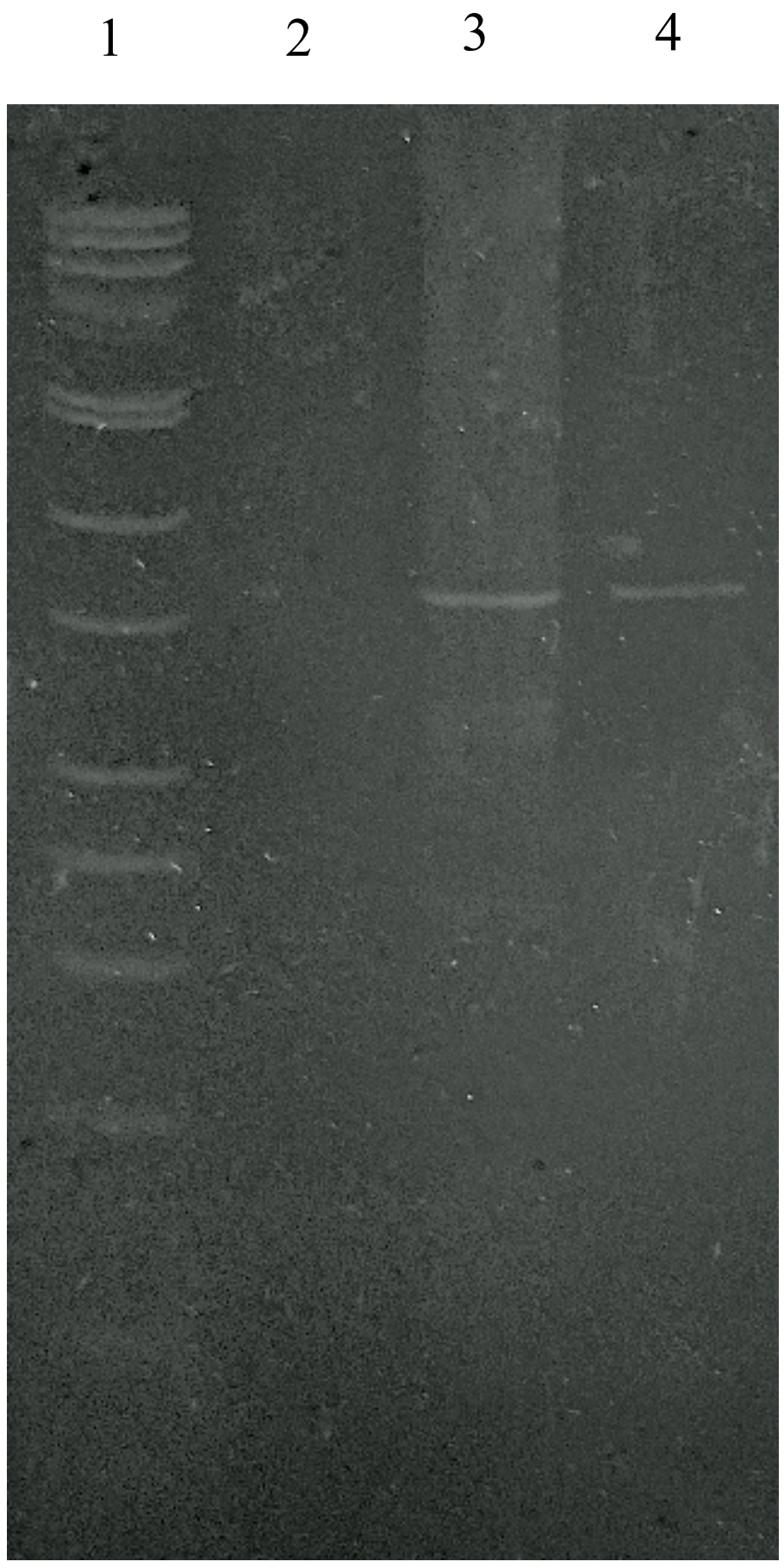

Supplementary Figure 2. Confirmation of recombinant vector transfection in *L. tarentolae*. Agarose gel electrophoresis (1.5%) results revealing the PCR products. Lane 1: Molecular weight marker (50–10000 bp); Lane 2: Amplification products of the neomycin resistance gene in wild-type *L. tarentolae* promastigotes (LtWT); Lane 3: Amplification products of the neomycin resistance gene in *L. tarentolae* promastigotes transfected with the recombinant vector (LtTRtar). Lane 4: Amplification product of the neomycin resistance gene in *L. tarentolae* promastigotes transfected with the pSP72αNEOα vector (LtPSP).

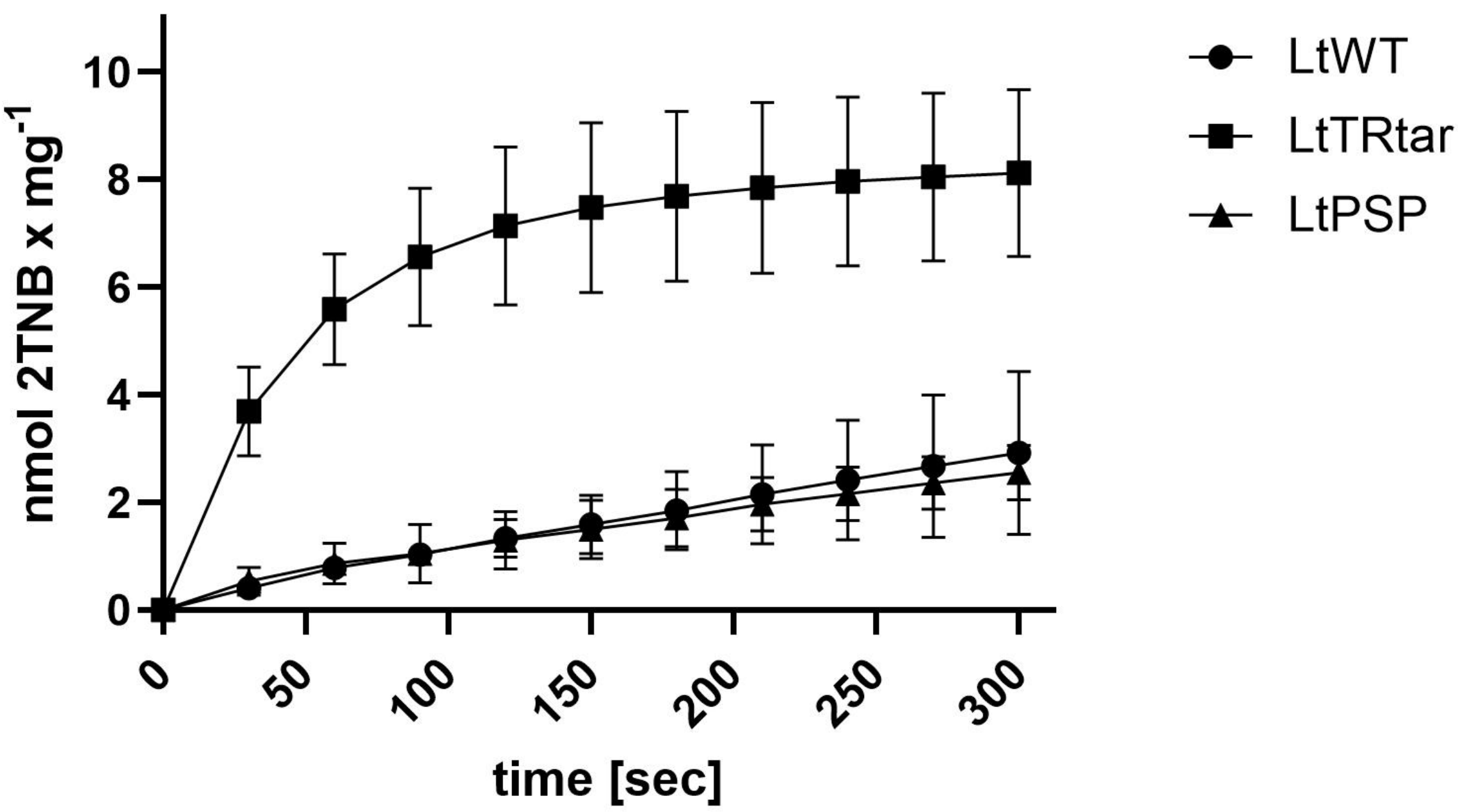

Supplementary Figure 3. Kinetic analysis shows stable, linear TR enzymatic activity in wild-type and TR-overexpressing *Leishmania tarentolae* promastigotes. Trypanothione reductase (TR) activity was measured by the Ellman reaction over 0 to 300 seconds. The soluble fraction was prepared from wild-type L. tarentolae (LtWT), L. tarentolae transfected with the empty vector pSP72αNEOα (LtPSP), and L. tarentolae transfected with pSP72αNEOα + TR of L. tarentolae (LtTRtar). The y-axis shows the amount of 2TNB (in nmoles per mg protein) produced during the reaction. Data are presented as mean ± standard error (n=3) from 3 independent experiments. The identical activity profiles of LtWT and LtPSP parasites confirm that the observed increase in LtTRtar (6-fold increase compared to LtWT and LtPSP) is specifically due to TR overexpression and not to vector effects. Statistical analysis was performed using one-way ANOVA with Tukey's post hoc test.
